# Medical Certificates in the Daily Papers

**Published:** 1878-02

**Authors:** 


### Medical Certificates in the Daily Papers.

The Committee on Ethics of the Medical Society of the County of New York made its first report on Monday evening last. Although this was merely of a preliminary character, there is enough of it to show that the members of the committee appreciate the responsibilities resting upon them, and that they are determined to do their duty. A proper initiative was taken in rebuking the writing of medical certificates in the daily papers, and in suggesting the only reasonable and suitable remedy for the growing evil, which is to the effect that "the offending parties shall at once withdraw their signatures from these advertisements, and thus place themselves in honorable record with this Society and the profession." This is the only course open to these gentlemen, one which we suggested some time since, and one which should have been taken by them of their own accord long ago, and when the certificates first appeared. As the case now stands, what would have been a matter of choice and ordinary prudence may become one of compulsion, which proceeding will carry a conviction upon its face. But the report is referred by rule to the Comitia Minora, which will doubtless take action at once, unless such may be made unnecessary by the prompt withdrawal of the names in question.—*Medical Record.*

